# Current Studies of the Effects of Drought Stress on Root Exudates and Rhizosphere Microbiomes of Crop Plant Species

**DOI:** 10.3390/ijms23042374

**Published:** 2022-02-21

**Authors:** Yalin Chen, Zongmu Yao, Yu Sun, Enze Wang, Chunjie Tian, Yang Sun, Juan Liu, Chunyu Sun, Lei Tian

**Affiliations:** 1 Key Laboratory of Straw Comprehensive Utilization and Black Soil Conservation, Ministry of Education, College of Life Science, Jilin Agricultural University, Changchun 130000, China; yalin12383@126.com (Y.C.); amuu0316@gmail.com (Z.Y.); tiancj@iga.ac.cn (C.T.); ysun@jlau.edu.cn (Y.S.); 2 Key Laboratory of Mollisols Agroecology, Northeast Institute of Geography and Agroecology, Chinese Academy of Sciences, Changchun 130102, China; sunyu08@iga.ac.cn (Y.S.); wangenze@iga.ac.cn (E.W.); 3 Sericultural Research Institute of Jilin Province, Jilin 132013, China; liujuan807@126.com

**Keywords:** microbiomes, plant, root exudates, drought stress, rhizosphere

## Abstract

With the warming global climate, drought stress is considered to be the most important abiotic factor limiting plant growth and yield in the world. Drought stress has serious impacts on crop production. Many researchers have studied the influences of drought stress on crop production and plant physiology; however, few researchers have combined root exudates with root-associated microbiomes for their mutual effects under drought conditions. In this review, we systematically illustrate the impact of drought stress on root exudates and root-associated microbiomes, and then we discuss the mutual regulation of root-associated microbiomes and the host plant in helping the plant adapt to drought. Finally, we construct a framework for the mutual connections between the plant, root exudates, and the microbiome. We hope this review can provide some significant guidelines to promote the study of drought resistance in plants in association with the rhizosphere microbiota.

## 1. Introduction

Water is the most important resource for plants, and plant organs need to maintain 60–90% water content for sustainable activity. However, global climate change, caused by greenhouse gas emissions, has become more serious worldwide, leading to drought throughout the world [1]. Drought has been considered the most serious and recurrent abiotic factor limiting crop growth and yield in the world [2]. Drought stress decreases metabolism in plants, and serious drought stress will cause electrolyte disturbances in plant cells, which will lead to death of the plant. Thus, sufficient water in the soil is vital for plant growth and production.

Currently, the frequent changes in climate have increased the severity of drought events for plants [3]. The plant-associated and plant rhizosphere microbiomes, including plant growth-promoting bacteria (PGPB) and plant growth-promoting fungi (PGPF), are closely related to plant growth and environment change, including drought. Drought stress influences plant water uptake, which inversely affects the plant metabolism and root exudates, and the exudates affect the plant rhizosphere microbiome. 

Furthermore, plant roots can help maintain the inherited microbial communities that may influence crop growth, nutrition, and health [4]. A previous study also showed that drought may produce legacy effects on soil microbial communities [5]. In contrast, the root-associated microbiome can help plants resist and adapt to drought. 

Although the composition of the root-associated microbiome has been determined in many crop species, the influence of abiotic stresses, especially drought stress, on the root-associated microbiota has been less studied [6]. In this paper, we review how drought stress affects plant root exudates and the root-associated microbiome, and then illustrate how the root-associated microbiome affects plants in resisting drought. We hope that this review can provide some significant guidelines for understating the mutual connections between plant root exudates and microbiomes under drought stress and can provide practice for developing beneficial microbiota in promoting plant resistance to drought stress.

## 2. Drought Stress Influences Root Exudates

Root exudates are plant metabolites and are secreted in the rhizosphere, including various compounds secreted or released from different parts of the root system into the rhizosphere environment during the growth of plants [7]. Root exudates can account for more than 10% of the plant photosynthate [8], including low molecular weight primary metabolites (especially sugars, amino acids, and organic acids) and secondary metabolites (phenols, flavonoids, and terpenoids) [7]. 

Root exudates, responding to the changing environment, can serve as positive or negative factors in plant growth [7,9]. A previous study demonstrated that drought promoted the secretion of organic acids in corn root, and malic acid was the main organic acid secreted [10], which may help to solubilize phosphate for plant utilization and promote drought resistance in plants. However, excessive root exudates will decrease the carbon storage in crop plants [11].

Root exudates serve as important carrier materials for material exchange and information transmission between plants and soil [12,13,14,15]. Root exudates are also the key factor to maintaining the vitality and function of the rhizosphere micro-ecosystem and are also an important part of the rhizosphere material circulation [12,13,14,15]. Root exudates can improve the bioavailability of soil nutrients and improve plant growth by changing the physical, chemical, or biological properties of the rhizosphere [12,13,14,15]. 

With the rapid development of modern instrumental analysis methods and the wide application of root exudates in the fields of plant nutrition, agroecological and environment studies, etc., the research on root exudates has entered a new upsurge. Especially in the last decade, with the construction and improvement of rhizosphere microecology, root exudates have been considered an important part of plant nutrition and rhizosphere microecology research.

Unlike animals, plants cannot escape various adverse environmental stresses and can only grow and develop in their germinating place where they finally complete their life cycle. Root secretion activity helps plants adapt to and gradually change the soil environment contacted by the roots, which is an adaptive response mechanism for plants to cope with environmental stress [12,13,14]. Therefore, the composition and amount of root exudates not only depend on the species and genotype of plants [16] but are also influenced and controlled by the external environment, such as drought. The composition and amount of plant root exudates under drought stress will also change to adapt to the stress [17]. 

In general, the content of root exudates of plants under drought stress will be higher than that under normal conditions. Many studies showed that the total content of root exudates (i.e., soluble sugars, amino acids, and organic acids) of the plants increased with the level of drought stress intensity [18,19]. The content of organic acids (malic acid, lactic acid, acetic acid, succinic acid, citric acid, and maleic acid) increased significantly in root exudates of maize under drought stress [18]. The increase in plant root exudates, especially organic acids, may help plants resist osmotic stress under drought stress.

Root exudates can improve the drought tolerance in plants. Drought stress will not only change the amount of plant root exudates but also significantly change their composition [8]. Gargallo-Garriga and others also studied the root exudates of *Quercus ilex* under gradient drought stress and subsequent recovery and found that the increased drought stress strongly affected the secondary metabolites (accounting for 71% of total metabolites), with the increased synthesis of alkaloids and terpenoids [20]. However, in the drought recovery stage, the composition of root exudates became dominated by primary metabolites (accounting for 81% of the total metabolites), and under extreme drought conditions, the changes in root exudates were irreversible, and plants could not recover [20]. 

Another study also showed that potassium (K+) and organic acid were the most important contributors to resisting drought stress in plants [21]. K+ is extracted from the soil by roots, where the roots can exchange it with organic acids [21]. Thus, with the frequent occurrence of drought now, we should not only strengthen research on the induction mechanism of specific root exudates under drought stress but also focus on the synthesis, transfer, and secretion pathways as well as the mechanisms of root exudates under drought stress. These studies may provide a theoretical basis for the development and utilization of plant resources in acclimating to drought stress.

## 3. Drought Affects Plant Hormone Production and Regulates the Metabolism

**Figure 1:**
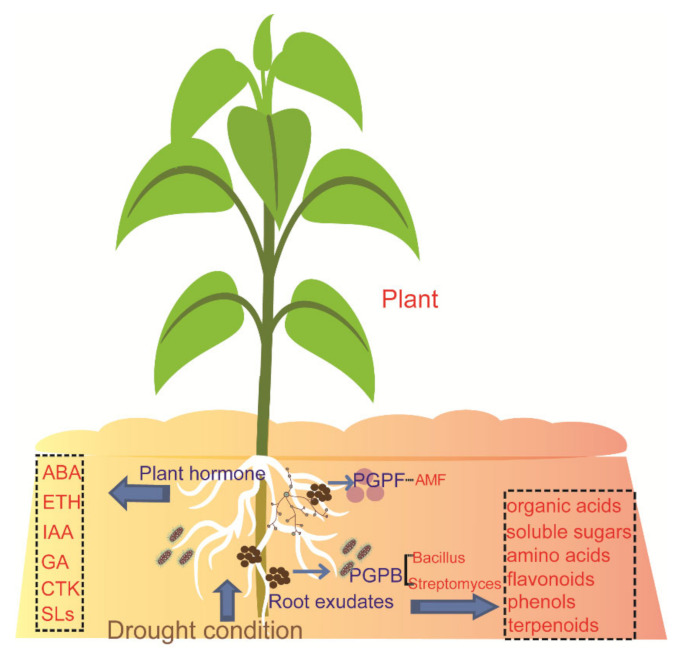
Drought stress influences root exudates and associated microbiotas. ABA, abscisic acid; ETH, ethylene; IAA, auxin; GA, gibberellin; CTK, cytokinin; SLs, strigolactones; PGPB, plant growth-promoting bacteria; and PGPF, plant growth-promoting fungi.

Plant hormones, which are produced in plants and transported from one place to another, play a regulatory role in the life activities of plants. The levels of endogenous hormones, abscisic acid (ABA), auxin (IAA), gibberellin (GA), cytokinin (CTK), and ethylene (ETH) are decreased or increased under drought (Figure 1) [22,23,24]. Many researchers have shown that, under drought conditions, the concentrations of ABA and ETH increase significantly [24,25], while it is less clear what happens to the concentrations of CTK, IAA, and GA. Studies showed that the concentrations of CTK, IAA, and GA decreased to a certain extent, and the endogenous hormones of varieties with strong drought resistance also changed greatly, which indicated that drought stress was negatively correlated with these hormone concentrations [26,27].

ABA is the most studied plant endogenous hormone under drought stress and is considered the most important hormone in plants for regulating signal transduction under drought stress [28,29]. Under drought stress, the plant roots receive the stress signal first, which stimulates the activity of ABA synthetase. Then, ABA, as a drought signal substance, is rapidly synthesized in the root cap and actively transported to the growth part, reducing stomatal conductance and water loss, thus improving the drought resistance [30]. Furthermore, plants can induce the expression of many genes under stress, and ABA can serve as the signaling molecule that induces the activation of these genes, which will lead to protein expression and other plant phenotypes [31,32]. 

In the potato budding stage, with the increase under drought stress, the ABA concentration and expression of the 9-cis-epoxycarotenoid dioxygenase (StNCED) gene (a key enzyme for ABA synthesis) in tubers showed an increasing trend. Under severe stress, the ABA concentration in tubers increased by 33%, and the transcription levels of the StNCED1 and StNCED2 genes increased by four and nine times, respectively [33]. Ding et al. (2016) studied the changes of endogenous hormones in rice seedlings under mild drought stress and concluded that the ABA concentration in rice root increased significantly with decreased precipitation [34].

It was shown that the concentration of strigolactones (SLs), one of the plant hormones, also decreases in plants under drought stress. However, evidence showed that SL levels increase in plants when faced with drought stress with the presence of arbuscular mycorrhizal fungi (AMF) spores adhering to the plant roots (Figure 1) [35,36]. SLs are also a signal substance for helping plants connect with AMF, and inoculated AMF helps plants resist biotic or abiotic stress. 

The dialogue between a plant and fungus comes from plant photosynthesis and root exudation, molecular cues (SLs), secreted into the rhizosphere [37]. It was shown that ABA can promote SL production under drought stress [38]. However, crosstalk analysis between cytokinins and SLs demonstrated that they have opposite effects on plant root drought adaptation, which indicated that CTKs act as negative regulators, while SLs act as positive regulators in responding to drought [36].

## 4. Drought Stress Influences Root-Associated Microbiomes Directly or Indirectly

Soil microbes can respond accurately and quickly to changes in soil environment, and small changes in soil environment can lead to changes in soil microbes, which can sensitively warn of small changes in terrestrial ecosystems. The soil water content is closely related to soil microbes, and drought will affect the rhizosphere microbial communities. First, drought will cause osmotic stress on soil microbes, which will directly affect them, resulting in their death and cell lysis. Second, drought will change the quality and quantity of carbon sources available to rhizosphere microbes by affecting plant photosynthesis and then indirectly affect the roots. 

Fuchslueger et al. (2014) demonstrated that water deficit caused a change in the microbial community composition; an increase of Gram-positive bacteria was driven by the drought stress, and drought decreased the C allocation below ground but did not influence the transfer of recently plant-assimilated C to fungi [39]. Comparative analysis of the effect of drought stress on the root and rhizosphere microbiomes of 18 species of monocot plants showed that a prominent enrichment in Actinobacteria during water deficit is common among the hosts [40]. Actinobacteria were more enriched in the root endosphere than in the surrounding soils [40], which indicated that Actinobacteria may be connected more closely with plant roots when facing water stress. 

Furthermore, the enrichment of Streptomyces (belonging to the Actinobacteria) under drought was shown to play a subsequent role in improving plant drought tolerance in the study [41]. Through the study of the interaction between soil components and wheat root microbiota under drought stress, it was found that the soil pH value and microbial biomass could significantly explain the changes in the microbial community, wheat genotype, and soil sodium and iron levels [42]. Under drought stress, the biodiversity in soil decreased significantly but increased in the rhizosphere community, in which specific soil parameters seemed to determine the enrichment of bacterial flora.

Drought stress leads to specific recombination of the rice root-related microbial community [6]. It was shown that enrichment of Actinobacteria and Chloroflexi, as well as depletion of several Acidobacteria and Deltaproteobacteria, was common for rice root-associated microbiota under drought stress. The study also showed that the plant genotype, as well as drought stress, influenced the root-associated fungal community in rice and that some fungi potentially improved plant drought tolerance [43].

In the soil environment, Gram-positive bacteria are more tolerant to drought than Gram-negative bacteria because their cell walls are thicker and some Gram-positive bacteria have the ability to form spores [41]. However, it has been observed that the enrichment degree of the Gram-positive bacteria in the relevant parts of plants is the highest, which indicates that the enriched Gram-positive bacteria described here are at least partly driven by the interaction within the plant host, not only by the ability of the Gram-positive bacteria to resist the dry environment [41]. 

Carbohydrates and amino acids produced by plants are powerful candidates among compounds that can promote the growth of a single germ layer [41]. It is well-known that root exudates have been demonstrated to selectively induce the growth of rhizosphere bacteria [44]. In addition, the recent work on the plant-related microbiome has confirmed that the correlation between the abundance of the Gram-positive bacteria (Actinobacteria, *Verticillium virens*, and Sclerotinia) and specific amino sugars and sugar alcohols, indicated that the Gram-positive bacteria are related to a group of different compounds, including polysaccharides and glycoside hydrolases. 

In addition, the metabolomics of roots under drought stress showed that the yields of many carbohydrates (including xylose and glucose) and amino acids (such as proline, threonine, and asparagine) increased significantly [45]. Malic acid, a well-known root exudate, is also an effective chemoattractant for *Bacillus subtilis* (e.g., in the soybean rhizosphere) when plants are under drought stress [10]. Santana et al. (2020) demonstrated that root exudates promoted the enrichment of *Bacillus* sp. ESA 402, one of the plant growth promoting bacteria, in the rhizosphere of sorghum (*Sorghum bicolor*), which benefited the growth of the sorghum under drought stress [46]. However, how the connection between the Gram-positive bacteria and the root exudates helps plants resist drought stress has not been clearly illustrated.

As for rhizosphere microbiotas, some scholars have used the dilution plate counting method to study the number of bacteria [40]. They found that fungi and Actinobacteria in the rhizosphere soil of ginger (*Zingiber officinale*), sorghum (*S. bicolor*), corn (*Zea maize*), wheat (*Triticum monococcum*), and others decreased with an increased level of drought stress intensity, while the number of bacteria and Actinobacteria in rhizosphere soil of cherry increased with the initial stress intensity and the decreased with a high level of drought stress [40]. 

Santos-Medellín et al. (2021) also showed that Streptomyces were the most enriched Actinobacteria in the rice root microbiome after drought stress [5]. Zhang et al. (2021) also showed that drought stress increased the diversity and abundance of the rhizosphere bacteria in both wild (*Glycine soja*) and cultivated (*Glycine max*) soybean [47]. However, the number of fungi decreased under drought stress in both studies [5,47]. Therefore, drought has a significant impact on soil microorganisms, and proper (mild or moderate) drought is beneficial for maintaining microbial diversity in rhizosphere soil [5,47]. Drought stress changes the composition of bacteria and fungi in rhizosphere soil, making bacteria dominant and then leading to the decline of the quality of organic substances. 

The ratio of fungi to bacteria is an important index for measuring the microbial community structure, which also reflects the nutrient status of the substrate [48,49]. The fungal pathway mainly occurs in soil with low nutrition, difficult decomposition, and a high carbon–nitrogen ratio, and the substrate circulation time is relatively long. This indicates that drought will have a great impact on the microbial community structure diversity of rhizosphere soil, and a certain intensity of drought can improve the microbial community structure diversity of rhizosphere soil, while excessive drought will lead to a decrease in the microbial community structure diversity of rhizosphere soil.

## 5. Root-Associated Microbiomes Regulate Plants in Resisting Drought Stress

Studies showed that not only did drought influence root-associated microbiomes but, conversely, root-associated microbiomes regulated plants in resisting drought stress. Plant growth-promoting rhizobacteria (PGPR) can not only help promote plant growth but also help plants resist biotic and abiotic stress [50]. It was also shown that PGPR can alleviate plant damage by inducing drought-responsive genes, such as the aquaporin (TaTIP1;1) and helicase genes [51]. Furthermore, plants contain various other resistance strategies to adapt to drought stress. 

In order to effectively absorb water and reduce water evaporation under drought conditions, the plant will decrease the leaf size and extend the root system into deeper soil [51,52]. Plants also benefit from the synthesis of different compounds for osmotic regulation (such as proline, glycine, betaine, and potassium), plant hormones (such as abscisic acid, salicylic acid, auxin, and gibberellin), and antioxidation (such as polyamine) when facing drought stress [52,53]. 

Although these strategies are promoted by plants, the contribution of bacteria also affects this protection. For example, although proline serves as the most vital osmotic agent in the plant cytoplasm, under environmental stress, proline accumulation occurs not only in higher plants but also in bacteria, which may also help the bacteria for drought tolerance and then in association with plant in resisting drought [54,55]. 

Rolli et al. (2016) showed that improved plant resistance to drought is promoted by the root-associated microbiome as a water stress-dependent trait [56]. The study also showed that the rice genotype, as well as drought stress, affected the root-associated fungal community, and conversely, some special fungi were shown to help improve the drought tolerance of rice [43]. The rhizobacteria can also produce plant hormones (such as IAA and CTK) or inhibit plant hormone production, which may directly affect plant growth [57,58]. 

The mechanisms of the plant in responding to drought stress by the rhizobacteria have been shown to include the production of abscisic acid, cytokinin, indoleacetic acid, trehalose, 1-aminocyclopropane-1-carboxylate (ACC) deaminase, volatile organic compounds, and exopolysaccharides [59,60]. The most studied beneficial fungi, arbuscular mycorrhiza fungi (AMF), are one of the most widely distributed fungi in the ecosystem, and they play an important role in the material circulation and energy flow in nature [61,62,63]. AMF can form mycorrhiza with host plants, and more than 80% of terrestrial plants have mycorrhiza [64,65]. 

They can also promote the plant absorption of nitrogen, phosphorus, and other elements in the soil, while plants can provide AMF with carbon sources generated by photosynthesis [65,66,67]. Rice, corn, wheat, soybean, and so on, which are the major food crops for human survival, are all mycorrhizal plants [68,69,70]. AMF not only forms a symbiotic relationship with rice in loose and breathable soil but also forms a symbiotic relationship with rice under flooded conditions [71,72,73]. Pavithra et al. (2018) confirmed that AMF inoculation can improve soybean biomass and proline content under drought conditions [74]. Drought may also improve the vesicle formation and hyphae development of AMF root colonization [75]. 

It is also shown that the extraradical hyphae of AMF colonized in roots extend into the soil. The extended and dense hyphae in the soil can contact and extract water from the soil pores, which is not accessible to plant roots and the root-hair zone [76]. As a strictly symbiotic fungus, AMF often forms nutrition complementation with plants while forming a symbiosis with them, and AMF plays important roles in promoting plant resistance to biotic and abiotic stresses [77,78,79]. AMF can improve trehalose in rice at low temperatures and help rice cope with low temperatures [73]. 

Trehalose, as an important non-reducing sugar, can not only be used in plant energy storage but can also be of great significance in assisting plants to cope with drought and low temperature [80]. Furthermore, when AMF hyphae infect plants or occupy favorable ecological positions, AMF can often inhibit the infection of other fungal hyphae to plant roots [81]. It can be speculated that AMF has a better effect on improving drought stress resistance of wild rice, and AMF infection can significantly improve the expression of plant-related genes and the ABA hormone level of wild rice [82]. Researchers demonstrated that the root exudates, strigolactones, and flavonoid regulated plant–AMF interactions can alleviate drought stress in plants [2,83].

## 6. Framework of the Mutual Connection among Plant, Root Exudates, and Associated Microbiota

**Figure 2:**
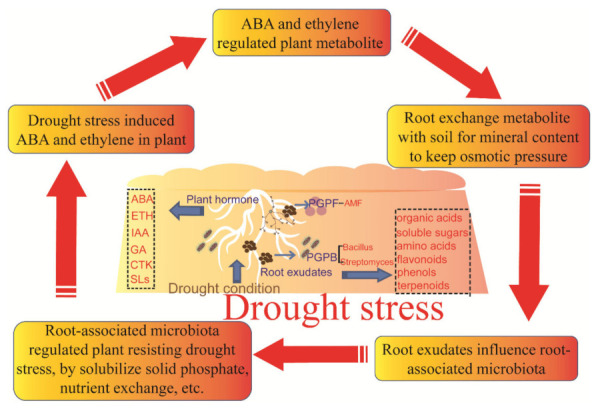
Framework of the mutual connection among plant, root exudates and associated microbiota. ABA, abscisic acid; ETH, ethylene; IAA, auxin; GA, gibberellin; CTK, cytokinin; SLs, strigolactones; PGPB, plant growth-promoting bacteria; and PGPF, plant growth-promoting fungi.

Based on the review and illustration of the studied and current understanding of the relationship among the plant, root exudates, and microbiome, we constructed a framework for mutual connections among them under wild and moderate drought conditions (Figure 2). Drought stress induces ABA and ethylene production, and the increased level of plant hormones regulates the plant phenotype and gene expression, which will decrease the plant metabolism and photosynthesis. 

The root cell extracts will contain more mineral material to keep the osmotic pressure by exchanging cell metabolism outside. Then the root exudates will change the root-associated microbial community structure, such as by increasing the abundance of Streptomyces. Then, the enriched PGPB or PGPF can stimulate the mineral extraction, which will help to regulate drought resistance in plants. 

However, there are also questions about the beneficial exchange of the root exudates for mineral between the plant root and soil microbes as well as the balance of the framework under the drought condition. Thus, in future studies, several aspects in connecting the rhizosphere microbiomes and root exudates of crop plants under drought conditions should be focused on: (1) the drought condition influence on root exudates, including primary and secondary metabolites; (2) the plant hormones secreted from the roots that regulate root-associated microbiota directly or indirectly; and (3) the crop plant exchange through the root exudates with the soil for mineral elements with the help of PGPB and PGPF.

## 7. Conclusions

In summary, drought stress has been shown to seriously influence root exudates and root-associated microbiomes in previous studies. Drought stress influences root exudates, including sugars, amino acids, flavonoids, hormones, etc. This stress not only influences soil microbiomes but also seriously affects rhizosphere communities, which may help plants resist drought stress. However, the crosstalk of the root-associated microbiomes with the root exudates has not been clearly illustrated. Thus, further studies should be focused on this topic in the future as this will have theoretical and practical significance for crop production.

## Data Availability

Not applicable.
